# Regulatory Perspectives in Precision Oncology: Challenges and Opportunities for Tyrosine Receptor Kinase (NTRK) Inhibitors in Non-small Cell Lung Cancer (NSCLC)

**DOI:** 10.7759/cureus.112577

**Published:** 2026-07-13

**Authors:** Justin Gainor, Sanjay Popat, Leah Bundy

**Affiliations:** 1 Center for Thoracic Cancers, Massachusetts General Hospital, Boston, USA; 2 Lung Unit, Royal Marsden Hospital, London, GBR; 3 Medical Education: Writing, Springer Health+ IME, London, GBR

**Keywords:** biomarker testing, non-small cell lung cancer, ntrk fusion, podcast, regulatory pathways, trk inhibitor, tumor-agnostic therapy

## Abstract

Neurotrophic tyrosine receptor kinase (*NTRK*) gene fusions are rare but actionable oncogenic drivers in non-small cell lung cancer (NSCLC). Despite the rarity of *NTRK* fusions, tumor-agnostic tropomyosin receptor kinase (TRK) inhibitors have demonstrated rapid and durable responses across multiple tumor types.

In this podcast interview, Justin Gainor and Sanjay Popat discuss the opportunities and challenges associated with developing and implementing targeted therapies for ultra-rare molecular subsets, such as *NTRK* fusion-positive NSCLC, from a regulatory perspective. Key topics include the evidence leading to the approval of TRK inhibitors, strengths and limitations of basket trials, reliance on single-arm studies and surrogate endpoints, differences between US and UK regulatory and reimbursement frameworks, and the impact of these factors on patient access. The discussion also highlights the critical role of comprehensive genomic profiling, as limited biomarker testing remains a major barrier to identifying eligible patients for TRK inhibitors. Potential solutions include greater regulatory flexibility, integration of real-world evidence, innovative approaches to evidence generation, and broader implementation of comprehensive molecular testing to support equitable access to precision oncology therapies.

## Editorial

This submission is a transcript of an interview between two expert oncologists (Video 1).

**Video 1:** Regulatory perspectives in precision oncology: challenges and opportunities for TRK inhibitors in NSCLC TRK: tropomyosin receptor kinase; NSCLC: non-small cell lung cancer

Chapter 1. Introduction

Justin Gainor (00:00:04:15 - 00:00:37:15): Welcome to our podcast entitled ‘Regulatory perspectives in precision oncology: challenges and opportunities for TRK inhibitors in non-small cell lung cancer’. My name is Dr. Justin Gainor. I am the Division Chief for Solid Tumor Oncology at the Massachusetts General Hospital and Mass General Brigham Cancer Institute, and an Associate Professor of Medicine at Harvard Medical School. It is my pleasure to introduce my colleague, Dr. Sanjay Popat, and let him introduce himself to the audience as well.

Sanjay Popat (00:00:39:03 - 00:00:51:21): Hi colleagues, and hi Justin. My name is Sanjay Popat; I am a Medical Oncologist at the Lung Unit at the Royal Marsden Hospital in London, England. And I am also a Professor of Thoracic Oncology at the Institute of Cancer Research, again in London.

Justin Gainor (00:00:54:09 - 00:01:25:13): Thanks so much for joining me. This is obviously an exciting topic, and we will kick things off with an introduction for our audience on neurotrophic tyrosine receptor kinase (*NTRK*) fusions. Obviously, the field of lung cancer has changed dramatically over the last 15 years, with the recognition that lung cancer is not just one disease; it is really a distribution of various oncogenic drivers.

Justin Gainor (00:01:26:17 - 00:02:05:13): We now have regulatory approvals for so many different targeted therapies. And this has transformed management of the disease for many of our patients. *NTRK* fusions tend to be amongst the rarest. We sometimes say in our clinic that maybe you find one or two in a career, but these patients are out there, and they really benefit from targeted therapies. In some of the retrospective series, it looks like a 0.1% or 0.2% prevalence for *NTRK* fusions in non-small cell lung cancer (NSCLC) [1].

Justin Gainor (00:02:07:09 - 00:02:59:12): The nomenclature here can be a bit confusing. So *NTRK* is the name of the gene. *NTRK* *1*, *2*, and *3* are distinct genes. The corresponding proteins are TRK A, B, and C [1]. And importantly, these drivers are mutually exclusive from others like epidermal growth factor receptor (EGFR) and anaplastic lymphoma kinase (ALK). We also know that patients with *NTRK *fusions really have distinct clinical pathologic characteristics. We tend to see these in adrenal carcinoma, never smokers, much like we do for many of our other targetable oncogenic drivers. Critically, they confer sensitivity to targeted therapies. Sanjay, can you tell us about what you expect to see with these agents in clinic?

Chapter 2. Overview of *NTRK* inhibitors in NSCLC and clinical evidence

Sanjay Popat (00:03:00:21 - 00:06:23:18): Thanks, Justin. The field of oncogenic disease has been transformed by the binding of oncogenic drivers. EGFR was the first one in 2004, and targeting a driver has been shown to cause rapid and durable tumor shrinkage. That has really stood the test of time with all the drivers that we've identified, particularly when it comes to *NTRK* fusions in *NTRK* 1, 2, and 3. I think understanding that these are three different genes with different fusions is important because people often think NTRK is one gene. It is important because, initially, we had two targeted agents, kinase inhibitors directed against the NTRK fusion, initially larotrectinib, and around the same time, entrectinib [2,3]. More recently, we have had the approval for repotrectinib [4]. These are oral therapies, these are kinase inhibitors, these are the tablets you take. Due to the rarity of the patients, the data that support them have been pulled from phase one studies. With larotrectinib, we've had the adult phase one trial, the pediatric SCOUT trial, the NAVIGATE trial, and basket trial of patients with many diverse diseases, many different tumors, all of which have the common finding of oncogenic fusions in *NTRK* 1, 2, and 3 [2,5]. And what Dr. Drilon and colleagues initially reported was rapid and durable responses, with responses occurring in up to 80% of patients, lasting beyond two and a half years on average [5]. With entrectinib, we see very similar data. Again, this was pulled analysis from the ALKA-372-001 phase one trial together with the STARTRK-1 and STARTRK-2 phase two trials [3,6]. Response rates are similar, maybe numerically slightly lower at around 60% [6]. Progression-free survival (PFS), also numerically slightly lower at around a year or so [6], but still very effective therapy. However, we know that we always get acquired resistance with kinase inhibitors, and this is a problem that we have [7]. Repotrectinib represents a next-generation NTRK inhibitor designed to access the binding pocket to overcome resistance. Clinical data on repotrectinib show a high response rate of around 60%, with very rapid responses [4]. In TKI-naive patients, this is very durable, lasting for over two years or so. But what is important is activity in patients with on-target resistance, for example, due to gatekeeper or solvent front mutations. In the solvent front mutation group, we see responses around 60%, with a duration response of approximately seven months [4]. So, this is a highly active agent.

Justin Gainor (00:06:25:12 - 00:07:30:15): I think that is a fantastic overview of the clinical data. It really highlights that the development of NTRK inhibitors was not done in isolation. It built on the history and the biology that we have come to understand for oncogenic fusions. We already knew about *ALK* and *ROS1 *fusions, right? We know that in the setting of these chromosomal rearrangements, this leads to constitutive kinase activation as well as oncogenic transformation in this state of oncogene addiction. That is what has enabled us to develop targeted therapies and deploy them in clinic, despite their rarity. I think the other thing that gets a lot of attention, and appropriately so with *NTRK*, is the implications it has had in terms of drug development.

Chapter 3. Tumor-agnostic drug development: benefits and challenges

Justin Gainor (00:07:31:18 - 00:10:14:21): As two thoracic oncologists, we tend to focus on lung cancer. However, one of the noteworthy aspects of *NTRK* as a driver is that it occurs not just in lung cancer; it occurs rarely in many common tumor types, and it is more common in some super-rare tumor types. This has really spawned this idea that when you are developing an agent for such a rare molecular alteration - and you've already alluded to this idea of kind of basket trials - where you take patients regardless of tumor type, who have a given alteration and are eligible for a study [8]. And so this idea of tumor-agnostic clinical trials, as well as tumor-agnostic regulatory approvals, was really one of the first examples of tumor-agnostic approaches. One of the benefits of this approach is speed and feasibility [8]. If we had tried to do an *NTRK*-specific trial in lung cancer, we would still be waiting for the data. This approach allows us to test the activity of these agents in a rapid way, in a very pragmatic way. It can also give us biological insights into the driver itself [8]. If we are seeing high response rates across tumor types, we recognize this as a shared vulnerability across tumor types. That is another positive aspect of these basket studies. And then lastly, it also gives us the ability to look at safety in a larger patient pool and across disease types. That is another benefit of the basket approach. Sanjay, do you think there are limitations of this approach or limitations of the data that we have thus far?

Sanjay Popat (00:10:14:21 - 00:12:57:11): One of the problems that we have with this approach is really understanding the granularity of what the NTRK-targeted drug is bringing to the disease in the wider context. We know what it is doing to the individual, but it becomes very difficult to understand what is going on across the overall group [8,9]. For example, if I have a group of nearly 30 different tumor types, ranging from adult tumors, mesenchymal tumors, epithelial tumors, lymphoblastic tumors, and pediatric tumors, where do I bring this drug into the patient's paradigm? Is this the first treatment that they receive? Do I use this in a curative setting or in late metastatic, last-line care, or somewhere in between? And I think it is that level of granularity that becomes a problem. The heterogeneity of the study populations is particularly problematic [8,9], particularly when you have pediatric patients on one side of the argument and elderly patients with lots of comorbidities on the other. The other issue is - so, look - you get very good tumor shrinkage, and you get shrinkage for a reasonable period of time. And for that period, this data needs to be in the public domain, right? Because it is making a difference. But of course, we want to know what longer-term impact it has on patients, both in terms of efficacy and safety. So, is it really making a difference on survival? Are people living longer as a consequence of this new therapy? You would think they probably are because it is a highly effective therapy, and it is keeping the cancer under very good, durable control for a reasonable period of time. But is it making that much difference, particularly when some tumor types may be very sensitive to other types of treatment? Now, whether you would rather have a TRK inhibitor or chemotherapy, I know what I would rather have. But there is an argument to be made at the population level, in terms of the payer’s viewpoint, in how that is perceived. Then, we need to consider that the data that we have are essentially on surrogate endpoints because the gold standard is overall survival, and we are never going to be able to do randomized studies in this setting. So these are the issues we need to think about when we are interpreting these studies. But Justin, I imagine these concerns are going to cause issues along the regulatory pathway, as regulators begin to consider this.

Chapter 4. Regulatory pathways and reimbursement considerations

Justin Gainor (00:12:58:21 - 00:14:31:08): That is a great segue because, practicing in two different regions of the world, I think it does highlight some differences. I can weigh in on the US perspective, and I would love your thoughts on the UK perspective. As I already alluded to, there were a couple of features here. One was the unmet need. Prior to this, patients with TRK fusions, particularly with lung cancer, were largely treated with chemotherapy and checkpoint inhibitors. And I think that the FDA took a very progressive view here of this idea of tumor-agnostic approval. The basket studies, they were quite small, right? The approval was really anchored on objective response and durability of response [2,3,5,6,10], to the point you were making earlier about different tumor types and some of the uncertainty, rather than time-to-event endpoints like PFS or overall survival, given the heterogeneity. And post-approval, there were some post-marketing commitments, but notably no requirement for a randomized study [10], which I think was appropriate. We would still be waiting. But maybe you can give us some of the UK perspective.

Sanjay Popat (00:14:32:14 - 00:16:28:20): In the UK, and in Europe more generally, we have a regulatory process and then a reimbursement process. I think that is slightly different from the US, right? Because in the US, the regulatory process is more closely aligned with reimbursement. From a UK-specific perspective, the UK is now outside the European Union from a regulatory standpoint [11]. Before Brexit, we were reliant on the European Medicines Agency (EMA), so companies would submit their dossier to the EMA, which would then review the risks and the benefits. If the drug was granted approval, it would be granted from EMA and that would be extended to the UK [11]. Post-Brexit, the UK has its own competent authority, the Medicines and Healthcare products Regulatory Agency (MHRA), which is now able to issue a marketing authorization for a drug, like those targeting NTRK. Now, the key issue is how do you enable approval in the setting of a rare disease? There are several ways that might happen. If you have a drug that is particularly promising, that is looking very effective as we have seen with these agents, there is now the opportunity post-Brexit to align with the U.S Food and Drug Administration (FDA). The FDA has a program called Project Orbis, which allows the regulatory FDA submission to be made to other partner regulatory authorities in different countries. The UK is one of several of these, along with others such as Swiss Medic [11,12]. This allows a much more rapid approval in the UK setting.

Sanjay Popat (00:16:29:20 - 00:18:45:06): The manufacturer may decide, for whatever reason, that Project Orbis is not the route they want to go down. There is what we call a direct submission mechanism, so the manufacturer can submit through a number of different routes directly to the MHRA. There is also something called EMA reliance. So what might happen is the manufacturer will say, well, look, we are not going to go directly to MHRA, we are going to submit our dossier to the EMA, which would then issue their approval, and the UK approval would be reliant on the EMA approval. This is historically what used to happen. And in this setting, when we have small data sets, there is usually a specific post-authorization obligation. We have seen this in many single-arm studies. The data set may be based on 60-70 patients; the regulator grants a conditional approval and then says, well, that's fine, but you now either need to run a randomized study, which we know in these contexts is going to be impossible, or you need to get a larger cohort to continue to accrue this data with longer follow-up, so we can be more confident about where that response lies, what the duration of response is, what the adverse event profile looks like, and the relationship between the two. And after that, after the label, all the countries in Europe have a reimbursement step. And in the UK, specifically in England, this is a body called the National Institute for Health and Care Excellence (NICE), which determines the cost-effectiveness of using something called the incremental cost-effective ratio (ICER), which looks at the quality-adjusted life years gained from implementing the new therapy. Each country within the European area has its own health technology assessment (HTA) mechanism for assessing the cost-effectiveness of introducing that drug. I think that is slightly different to the US system, isn't it, Justin?

Justin Gainor (00:18:45:06 - 00:18:50:05): It is, and I think you draw an important distinction there. The regulatory approval in the US is not linked to cost-effectiveness. It really comes down to whether the drug is safe and effective, but you are right, I think that is a key distinction.

Sanjay Popat (00:19:14:19 - 00:21:35:09): And in the UK, there are several technical issues to consider. For example, when these drugs are evaluated by NICE, there is a lot of uncertainty driven by the longitudinal outcomes, and this uncertainty directly affects the cost-effectiveness assessment. That creates a problem because when we have more uncertainty around longer-term outcomes, there is consequently more uncertainty about what is considered cost-effective. This is particularly problematic for rare indications. For example, in the UK at the moment, entrectinib was originally approved in 2020 under a conditional funding agreement. In 2025, it came up for a re-review of funding. This year, the manufacturer did not provide an evidence base for that. As a result, NICE withdrew funding for entrectinib in *NTRK* fusion-positive tumours. When we look at the regulatory documents, in that 6-year period, only 12 cases were registered in the national database using entrectinib for *NTRK* fusion-positive cancers, highlighting the rarity of this population. For larotrectinib, the renegotiations after the five-year period are still ongoing. Larotrectinib was originally reimbursed in 2020, and publicly available data suggest that it has been used in around 60 cases in England for *NTRK* fusion-positive cancers. Again, this highlights some of the challenges we face with health economics in rare diseases. The other thing we need to think about as physicians is that the local health authority will also impose additional restrictions. For example, NICE may say that a drug is approved and funded for patients with Eastern Cooperative Oncology Group (ECOG) performance status 1, but NHS England will say that it is ECOG performance status 0-1, you can only use it in later lines of therapy, and you need to have patients with additional restrictions, such as adequate liver and haematological function requirements, and no active brain metastases. These restrictions often lead to inequities in access.

Chapter 5. Access to testing and treatment

Justin Gainor (00:21:36:24 - 00:22:39:02): Yeah, I think that is a great point and a nice segue. I would like to pivot a little bit to disparities in access to treatment. You just highlighted several of them, and this also starts with access to testing. Access to the drug is the second part, and access to testing is certainly a major barrier. We know that for lung cancer and many other tumor sites, to find *NTRK* fusions, there are a couple of different molecular tests [13]. But as you start screening for biomarkers, you cannot rely on single assays for each gene, right? Especially given the number of tests that need to be performed. So next-generation sequencing (NGS) tends to be the preferred modality, as it allows testing for all drivers simultaneously.

Justin Gainor (00:22:40:20 - 00:23:55:14): Fusions can be tricky to identify with some platforms. For *NTRK* in particular, it does suffer a bit from the rarity. You highlighted the number of cases identified since 2020. That certainly is a concern, but it can be framed in the context of, "Hey, we have to be testing for all these other alterations as well.” This really underscores that it is easy just to perform the broad molecular testing. Yet we have people still not getting tested. I recognize that it is multifactorial, right? Sometimes the tissue is not adequate [13], and we have to think about a repeat biopsy. But I also think that blood-based testing has made this much more straightforward, knowing that if there's not enough tissue, and if we find something in the plasma, we can certainly pursue treatment. Sanjay, are there other things that I am missing in terms of barriers to testing?

Sanjay Popat (00:23:55:14 - 00:26:07:15): I think you covered the main ones. And the rarity of *NTRK* fusions is a particular problem, right? Because people forget that it is relevant - that is one of the key issues. The plasma testing is an important one. In England, we are lucky enough to have a plasma first program. So when the patient hits the respiratory clinic or pulmonology clinic with suspicious symptoms or a suspicious computed tomography (CT) scan, we have funding to use the circulating-tumor DNA (ctDNA) test at presentation, which is very forward-thinking. But Justin, as you know, picking up *NTRK* fusions on the ctDNA test is challenging, right? Because of the technical aspects and chemistry of ctDNA. And as we know, RNA sequencing can be problematic as well, because you must have a certain amount of tumor sample [13]. Sometimes, the RNA fails because of processing issues. There are multiple reasons why the test may be funded, but, actually, the test is not done or completed adequately. These things take time as well. NGS takes time, so getting these results can be problematic. There are many different problems with this. We are trying to get reflex pathways to overcome these limitations. Luckily, we have adequate resources, but the coordination between the pathology laboratory and the molecular laboratory could be better. We also need to ensure that we have adequate genomic literacy. I do think one of the big issues is that people may not know that they have an *NTRK* fusion in that report. So adoption of RNA-based testing is really critical [14], having clear reports is critical, and adequate education for our oncologists. The wider clinical group is fundamental to ensure that we can get rid of all these different bottlenecks. What do you think the future is looking like, Justin, for *NTRK* and other biomarkers in lung cancer and other diseases as well?

Chapter 6. The future of precision oncology for rare biomarkers

Justin Gainor (00:26:08:22 - 00:28:29:18): I would split this into two aspects - the scientific aspect and the regulatory side. Biologically, I think as sequencing technologies evolve, with long-read sequencing and other advances, we will continue to make novel insights into new therapeutic vulnerabilities. As we make these insights, however, we are likely to find increasingly rare populations. So, we need to think more dynamically from a regulatory perspective, because we cannot do big randomized phase-three studies for all of these advances. Then, we must start thinking about whether it is possible to do single-arm studies, or if we can augment that in some way? Can we create synthetic control arms, digital twins, or can we leverage real-world data? All these things are on the table, especially as our health systems, particularly in the US, are increasingly integrated with our electronic medical records (EMRs). I would say that as the testing companies start collecting more outcomes data, it really does help facilitate real-world evidence generation. That is particularly important for alterations like *NTRK*, where a single institution may only have a few patients, but then you could look at a system-wide level, at a country-wide level, at a global level. That can really help with the evidence generation. But how does our regulatory structure also evolve as the biology evolves? Sanjay, what do you think about that? Now we have, as you mentioned, three TRK inhibitors, right? Is there a role for newer TRK inhibitors, and how do we evaluate them within a regulatory context? Now that we already have three approved agents, one could ask whether there remains an unmet need.

Sanjay Popat (00:28:30:19 - 00:31:24:19): We have an unmet need because patients are still dying of *NTRK* fusion-positive lung cancer, which is the bottom line. Our drugs are good, but they are not that good. We need better NTRK inhibitors with a better range of side effects that are a little more tolerable. And we need NTRK inhibitors that can overcome diverse mechanisms of resistance, both on-target and off-target, and we need to think about how we combine them with other drugs as well. But even more than that, we need to think about rare biomarker-driven subsets in cancer. How do we achieve regulatory reform? Because the regulatory system, maybe less so in the US, but fundamentally in Europe, is still very much based on randomized controlled trials. That is really what the regulators are looking at, but that is just not going to be possible. If I have one rare biomarker, which is doing wonderful things in a super-rare population, it still creates big issues with reimbursement. Our reimbursement system is also very traditional, relying on having something comparable where the cost is known over a longer period of time. We need to think a bit more flexibly. We need to think about accelerated approvals and be comfortable with single-arm studies to get the approval, and then generate wider data, as you have done with the FDA system. That is important in Europe, to allow a greater amount of flexibility when it comes to reimbursement. Some things are happening with the wider political agenda that we have seen globally. We have seen lifting of some thresholds by NICE, driven by the economic changes we have seen in the US recently. And we have seen difficulties with NICE and the UK environment. We used to have something called the end-of-life criteria, which lifted the quality-adjusted life years (QALY) threshold from 30K to 50K, allowing greater spend for some cancers, but that has been eradicated now. We have something called severity modifiers, which adjust the weight applied to QALYs, typically from 1.2 to 1.7. This is causing some problems because there is ongoing debate about which level of severity modifier applies to your particular disease. I do think there is still more work to be done. Hopefully, the HTA agencies and the regulatory agencies, alongside the clinicians, can coordinate a bit better. Justin, I would be interested in your thoughts on that.

Justin Gainor (00:31:25:22 - 00:32:27:04): I completely agree. I think this has been a great discussion. I concur that we need greater alignment between regulators, sponsors, clinicians, and testing companies. This conversation has really highlighted the importance of that, especially as we think about expanding targeted therapies to other oncogenic subtypes, and not just within lung cancer, but more broadly across oncology. This has been a phenomenal discussion. I want to thank our audience, and I want to thank Sanjay for joining and for his expertise.
